# A tree-ring δ^18^O based reconstruction of East Asia summer monsoon over the past two centuries

**DOI:** 10.1371/journal.pone.0234421

**Published:** 2020-06-09

**Authors:** Dai Chen, Feifei Zhou, Zhipeng Dong, A’ying Zeng, Tinghai Ou, Keyan Fang

**Affiliations:** 1 National Forestry and Grassland Administration, National Park Administration, Beijing, China; 2 Key Laboratory of Humid Subtropical Eco-geographical Process, Ministry of Education, College of Geographical Sciences, Fujian Normal University, Fuzhou, China; 3 Department of Earth Sciences, Regional Climate Group, University of Gothenburg, Gothenburg, Sweden; Woods Hole Oceanographic Institution, UNITED STATES

## Abstract

The East Asian summer monsoon (EASM) exhibits considerable decadal variations since the late 20th century. Efforts to examine long-term behaviors and dynamics of the EASM are impeded largely due to the shortness of instrumental meteorological records. So far, reconstructions of the EASM with annual resolution from its core regions remain limited. We conduct the first 200-year robust EASM reconstruction based on tree-ring cellulose δ^18^O records derived from *Pinus massoniana* trees growing in the middle Yangtze River basin, one of the core EASM areas. The δ^18^O chronology accounts for 46.2% of the actual variation in an index of the EASM from 1948 to 2014. The reconstructed EASM indicates that the monsoon intensity was below average before the 1950s, peaked in the 1950s-1970s, and then began to decline. The reconstructed EASM is negatively correlated with the El Niño-Southern Oscillation (ENSO), but this teleconnection is dynamic through time, i.e. enhanced (reduced) ENSO variability coheres with strong (weak) EASM-ENSO connections. In addition, despite high ENSO variability since the 1980s, the EASM-ENSO relationship weakened possibly due to anthropogenic impact, particularly aerosol emissions.

## Introduction

The Asian summer monsoon is the strongest monsoon system on Earth, influencing the hydroclimatic conditions of dozens of countries and livelihood of roughly half of the world’s population [1, 2]. The East Asian summer monsoon (EASM) is a subtropical monsoon encompassing East China, Japan and Korea [3, 4]. EASM reaches the northernmost location in the global monsoon system to semi-arid regions in eastern and central Asia, forming the ecotone between agricultural and animal husbandry. Abrupt shifts of EASM usually lead to anomalous hydroclimate changes in East Asia with devastating consequences for regional agriculture and socioeconomics [1, 5] and even alternation of dynasties in history [6, 7].

Proxy-based reconstructions of EASM have revealed positive responses of EASM to historical warming at centurial- or even millennial-long scales [8, 9]. However, under the recent global warming, the instrumental records indicated a decay of EASM [1, 10, 11]. It is generally believed that recent changes in EASM is strongly influenced by enhanced anthropogenic forcings [1, 10]. To evaluate the recent anomaly, a long-term background of natural EASM variability is needed. Although there have been some EASM reconstructions from lake sediments [9, 12], peat deposits [13] and stalagmites [8, 14], these archives are often unable to resolve interannual to interdecadal variations due to dating accuracy and coarse sampling resolution. This poses a challenge in deriving a robust calibration with the EASM index, hence high-resolution proxy records are needed.

Tree rings can provide exactly dated, annually resolved information about past climates [15, 16]. A growing body of tree-ring chronologies were published from the core areas of the EASM-affected regions (e.g. South and East China) [17, 18, 19, 20], but seldom were used to reflect EASM dynamics because tree rings in these warm and humid conditions are normally insensitive to hydroclimate changes [21]. Tree-ring cellulose oxygen isotopes (δ^18^O) usually contain information on source water isotope compositions and atmospheric vapor pressure deficit [22], which are closely related to regional precipitation δ^18^O [23]. Precipitation δ^18^O is negatively correlated with precipitation amounts due to the “amount effect” in the tropical monsoon environment [24, 25, 26, 27]. Therefore, oxygen isotope records preserved in tree-ring cellulose are potential archives of robust monsoonal hydroclimate signals in many warm-humid regions [28, 29, 30, 31, 32]. Moreover, the tree-ring δ^18^O preserve long-term climate trends, since it does not require trend removal, in contrast to tree-ring width data.

In this study, we built a δ^18^O chronology from *Pinus massoniana* trees in the middle Yangtze River basin, a core area of EASM [33], to derive the first reconstruction of EASM index covering the period from 1815 to 2014. Based on this reconstruction, we investigate the characteristics and regimes of EASM with a long-term perspective and identified possible processes that drive EASM over the past two centuries.

## Material and methods

### Ethics statement

All field work and sampling were carried out with official permission from Hubei and Hunan Forestry Bureau, China.

### Climate and tree-ring width data

**Fig 1:**
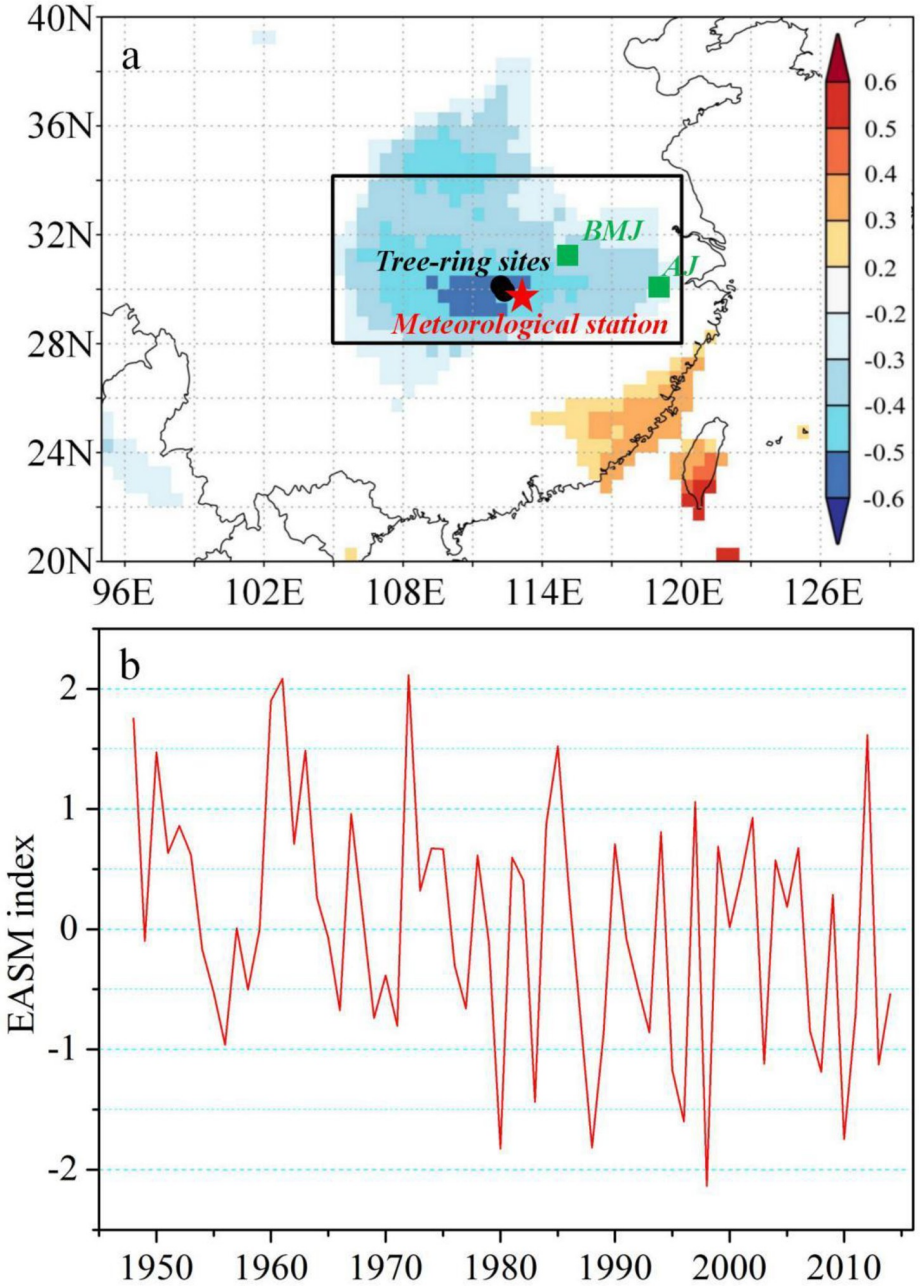
(a) Locations of the tree-ring sampling sites (circle), the meteorological station (star) and the other tree-ring δ^18^O based precipitation reconstructions (square) [30, 31]; background colors represent the spatial correlations between the observed EASM index [33] and CRU TS4.03 precipitation (from June to August); the black box highlights the study area; (b) The observed EASM index from 1948 to 2014.

The sampling sites (HP, 110.55° E, 30° N, 860m a.s.l.; WL, 110.67° E, 29.83° N, 893m a.s.l.), located in the middle Yangtze River basin, have a humid and warm subtropical monsoon climate (Table 1; Fig 1A). The instrumental records from the nearest Shimen meteorological station (111.37° E, 29.58° N, 198 m a.s.l.), obtained from China Meteorological Administration (www.nmic.gov.cn), reveal that the mean annual total rainfall from 1960 to 2014 is 1360 mm, the annual average temperature is 16.9°C and the mean relative humidity is 75.4% during the period of 1961–2016. The average total precipitation in monsoonal season (June-July-August) exceeds 580 mm, contributing more than 42% of the annual total precipitation. The monsoonal precipitation shows strong interannual variability, with the standard deviation exceeding 210 mm.

**Table 1:** Statistics of the two tree-ring sampling sites, the Shimen meteorological station and the CRU grid point.

Data type	Site code	Location (Longitude, latitude)	Elevation (m)	Number (core/tree)
Tree-ring	HPS	110.55° E, 30° N	860	36/18
	WL	110.67° E, 29.83° N	893	30/15
Meteorological data	Shimen	111.37° E, 29.58° N	198	

The EASM index (Fig 1B) used as a calibration data for reconstruction is defined as an area-averaged seasonally (June-July-August) dynamical normalized seasonality of wind fields at 850 hPa within the East Asian monsoon domain (10°-45° N, 110°-140° E) during the 1948–2014 period [33]. The index is available from the website (http://ljpgcesscn/dct/page/65577), which is widely used to show summer monsoon activity in the middle-lower Yangtze River basin [33]. In addition, monthly gridded precipitation from the Climate Research Unit (CRU) TS4.03 [34] and monthly sea surface temperatures (SSTs) during 1870–2014 from the Hadley Centre Sea Ice and Sea Surface Temperature dataset version 1.1 (HadISST1.1) [35] are used for climate-proxy relationship analysis. The spatial correlations between the EASM index and SSTs are used to investigate the teleconnections between EASM and large-scale atmospheric-oceanic circulations. The spatial correlation analysis are performed using the KNMI Climate Explorer (http://www.knmi.nl), a web-based application for high-resolution paleoclimatology [36].

In the fieldwork, two cores per tree were extracted using increment borers. In total, 66 increment cores were taken from 33 living *P*. *massoniana* trees at the sites (Table 1). The samples were mounted, air dried, polished and cross-dated based on the standard dendrochronology procedures [37, 38]. Ring widths were measured to 000.1 mm accuracy and quality checked with moving correlations using the COFECHA program [39]. The ARSTAN program [40] was used for the standard chronology development. As the sample size generally declines in the early portion of a tree-ring chronology, the subsample signal strength (SSS) statistic [41] with a threshold value of 0.85 is employed to evaluate the most reliable time span of the chronology. The final ring-width chronology exceeds the SSS threshold of 0.85 over the period 1815 to 2014 (S2 Fig).

### Cellulose extraction and oxygen isotope measurements

We selected eight cores from eight trees (5 cores from HP, 3 cores from WL) with no absent rings and homogeneous growth patterns (interseries correlations greater than 0.5) for stable isotopes analysis, covering a period of 1815–2014. We “pool” the whole wood from their annual rings prior to α-cellulose extraction following standard pooling methodology [42]. The “pooling” method has been proven to be reliable at our sites according to high coherency of tree-ring cellulose δ^18^O data of four individual cores from four different trees during the period of 1950–2014 (S3 Fig and S1 Table). Then α-cellulose of the annual tree rings were extracted following a modified method of [43] and [44]. In order to homogenize the cellulose, an ultrasonic water bath (JY92-2D, Scientz Industry, Ningbo, China) was used to break the cellulose fibers. The α-cellulose was then freeze-dried for three days prior to isotope analysis. The tree-ring δ^18^O values were measured using a High Temperature Conversion Elemental Analyzer (TC/EA) coupled to a Finnigan MAT-253 mass spectrometer (Thermo Electron Corporation, Bremen, Germany). Each cellulose sample was repeatedly measured four times to improve precision. The resulting standard deviation of the replicates was less than 0.3‰ based on the four measurements [24]. We calculated the mean isotopic values of each sample without outliers (values more than three standard deviations from the mean). We measured the ratio for a benzoic acid working standard (repeated four times) with a known δ^18^O value (IAEA-601, 23.3%) every seven measurements to monitor the analytical precision and to calibrate the samples for analytical accuracy [45]. The IAEA-C3 cellulose standard (δ^18^O = 3.22‰) was also employed here to calibrate the tree-ring oxygen measurements. The resulting standard deviation of the replicates was less than 0.3‰ based on four measurements.

### EASM reconstruction

EASM reconstruction was conducted by a simple linear regression model on the basis of a split calibration-verification procedure that was designed to test the model reliability [46]. A number of statistics were employed to evaluate model ability, including simple correlation coefficient (R), R square (R^2^), reduction of error (RE) and coefficient of efficiency (CE). Values of RE and CE greater than zero indicate rigorous model skill [16].

## Results

**Fig 2:**
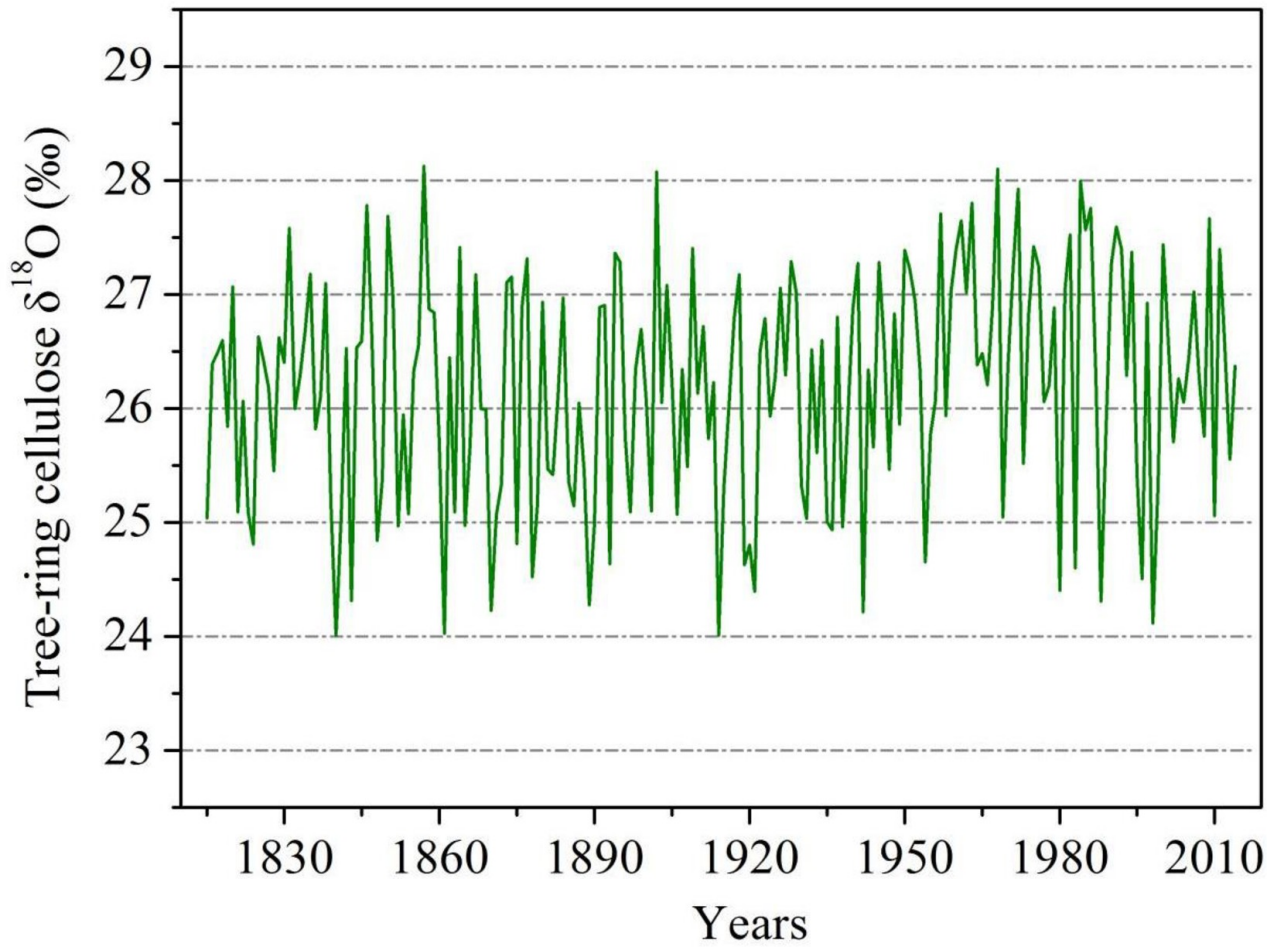
The tree-ring δ^18^O chronology during the period 1815–2014.

**Fig 3:**
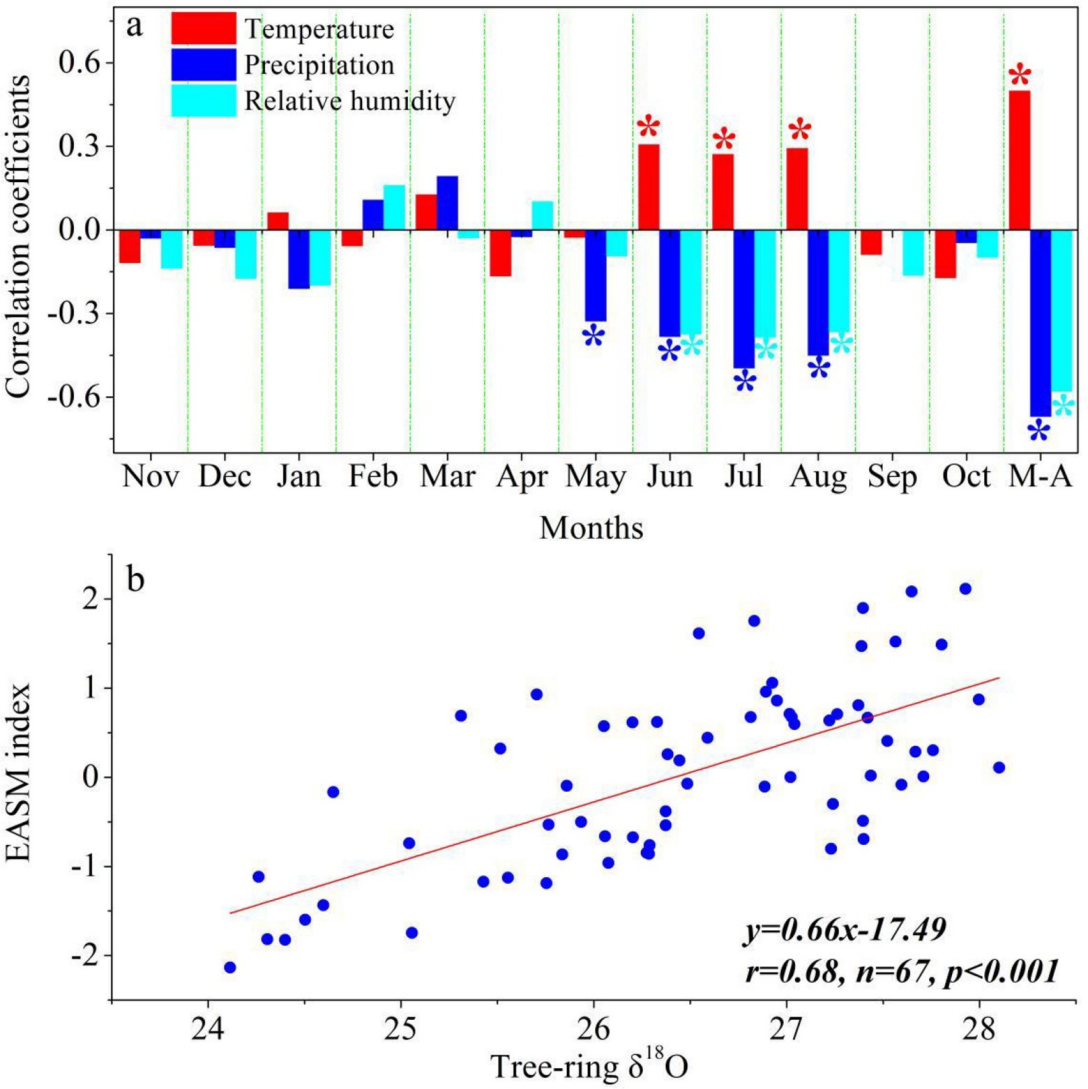
a) Correlations calculated between the tree-ring δ^18^O chronology and climate variables recorded by Shanghang meteorological station from previous November to current October during the period of 1960–2014. *M-A* symbolizes the combined months from May to August. Stars indicate the 95% confidence level; b) Relationship between the δ^18^O chronology and the observed EASM index for the period of 1948–2014.

Significant spatial correlations are found between the EASM index and summer precipitation from CRU TS4.03 in the middle-lower valleys of Yangtze River (Fig 1A), indicating that the tree-ring sites are located in the core regions of EASM. The composite tree ring δ^18^O chronology from *P*. *massoniana* trees at our sites is given in Fig 2. The mean and standard deviation of the δ^18^O time series are 26.19‰ and 0.99‰, respectively. The autocorrelation (one year lag) of the δ^18^O chronology is 0.15. Correlation analysis was performed between the tree-ring δ^18^O chronology and monthly climate variables (temperature, precipitation and relative humidity) from previous November to current October during the period of 1960–2014. The δ^18^O signatures correlated significantly (*P*<0.05, N = 55) and negatively with precipitation and relative humidity from May to August (Fig 3A). In addition, tree-ring cellulose δ^18^O records also show significant (*P*<0.05, N = 55) and positive correlations with temperature during the months of June, July and August. It appears that the hydroclimatic changes during the monsoonal season are the dominant limitations for tree-ring δ18O in the study area. The highest correlation (0.67, P<0.01, N = 55) with climate is observed for precipitation in the combined months from May to August.

**Fig 4:**
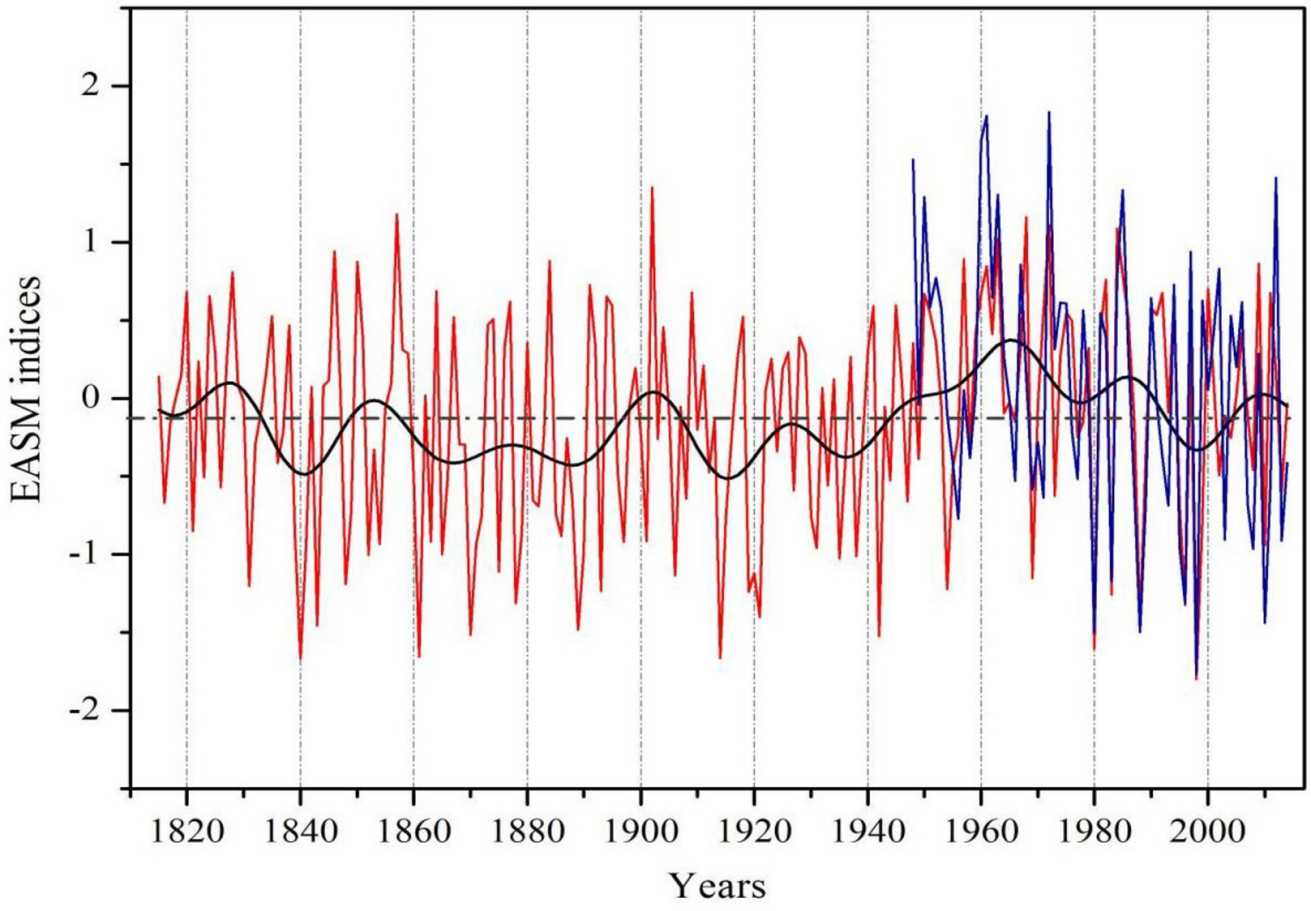
**The EASM reconstruction (red line) back to 1815, with the interdecadal variations (bold black line) isolated by using a 20-year fast-Fourier transform filter and the target time series (blue line) for the 1948–2014 period of overlap.** The horizontal dashed line represents the mean value of the reconstructed EASM over the period of 1815–2014.

Given close associations of tree-ring δ^18^O with summer monsoon rainfall, the correlation with the EASM index [33] was calculated for the 1948–2014 period. The high correlation (0.68, *p*<0.001, N = 67) between the EASM index and tree-ring δ^18^O indicates that the EASM index can be a strong predictand of variance in tree-ring δ^18^O in the study area (Fig 3B), and was selected as the target for reconstruction. The linear regression model accounts for 46.2% of the actual variance of EASM during 1948–2014. As shown in Table 2, the calibration and verification results for the split subperiods, i.e. 1948–1980 and 1981–2014, generally show a good model fit. Based on this model we reconstruct temporal changes of EASM for the past 200 years (Fig 4). Wavelet power spectrum analysis indicates that the reconstructed EASM contains interannual (<10 years) variations at a confidence level greater than 95% (S5 Fig).

**Table 2:** Statistics of the split calibration-verification model for the EASM reconstruction.

	Calibration (1948–1980)	Verification (1981–2014)	Calibration (1981–2014)	Verification (1948–1980)	Full calibration (1948–2014)
R	0.66**	0.71**	0.71**	0.66**	0.69**
R^2^					0.48
RE		0.44		0.52	
CE		0.49		0.39	

RE: reduction of error; CE: Coefficient of efficiency

** represents the the 99% confidence level

**Fig 5:**
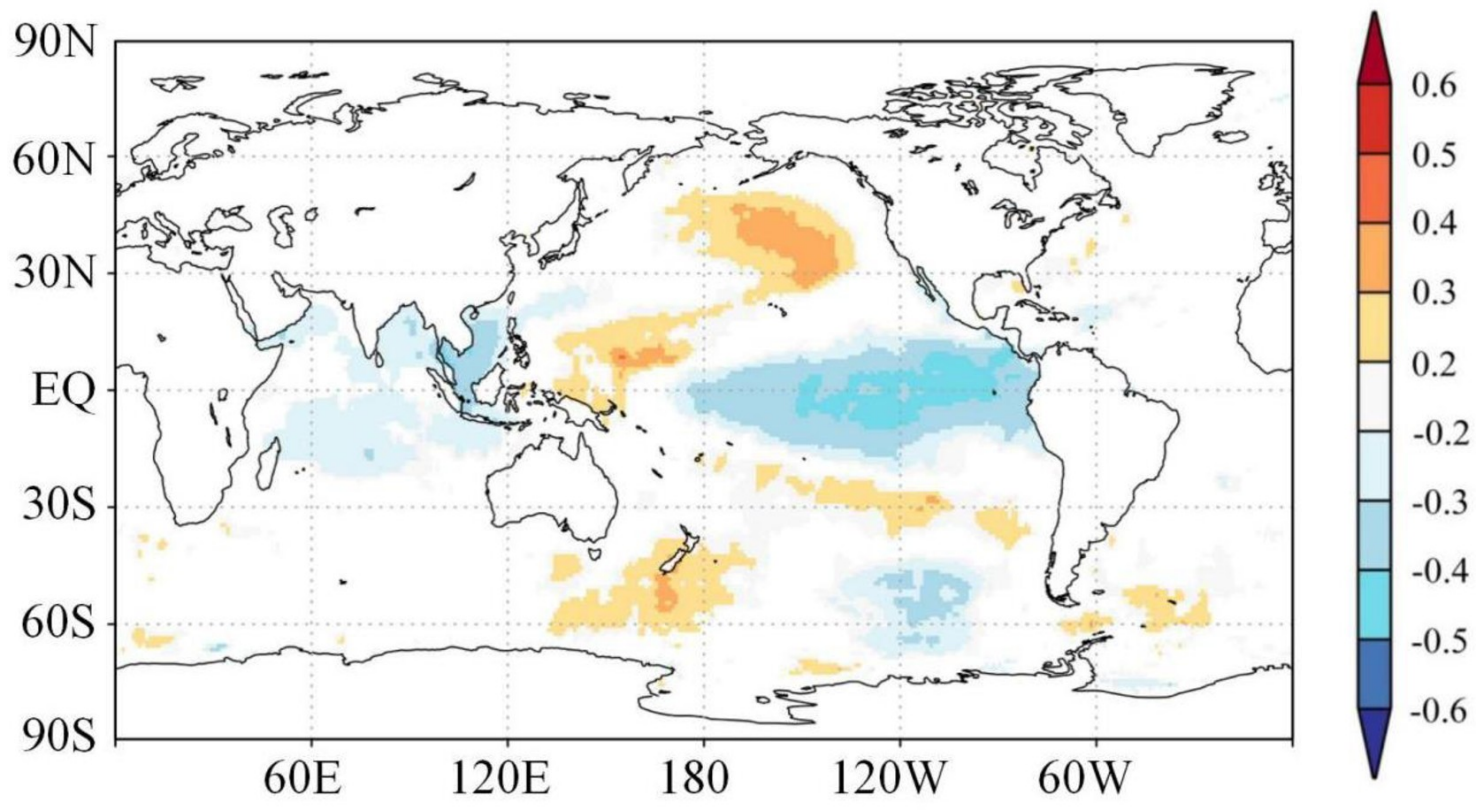
Correlations between the reconstructed EASM and precedent winter (from December to February) global SSTs for the period of 1871–2014. Correlations not significant at the 95% level have been masked out. *The maps were produced with GrADS v2.2.0 software (http://cola.gmu.edu/grads/) in KNMI Climate Explorer*.

**Fig 6:**
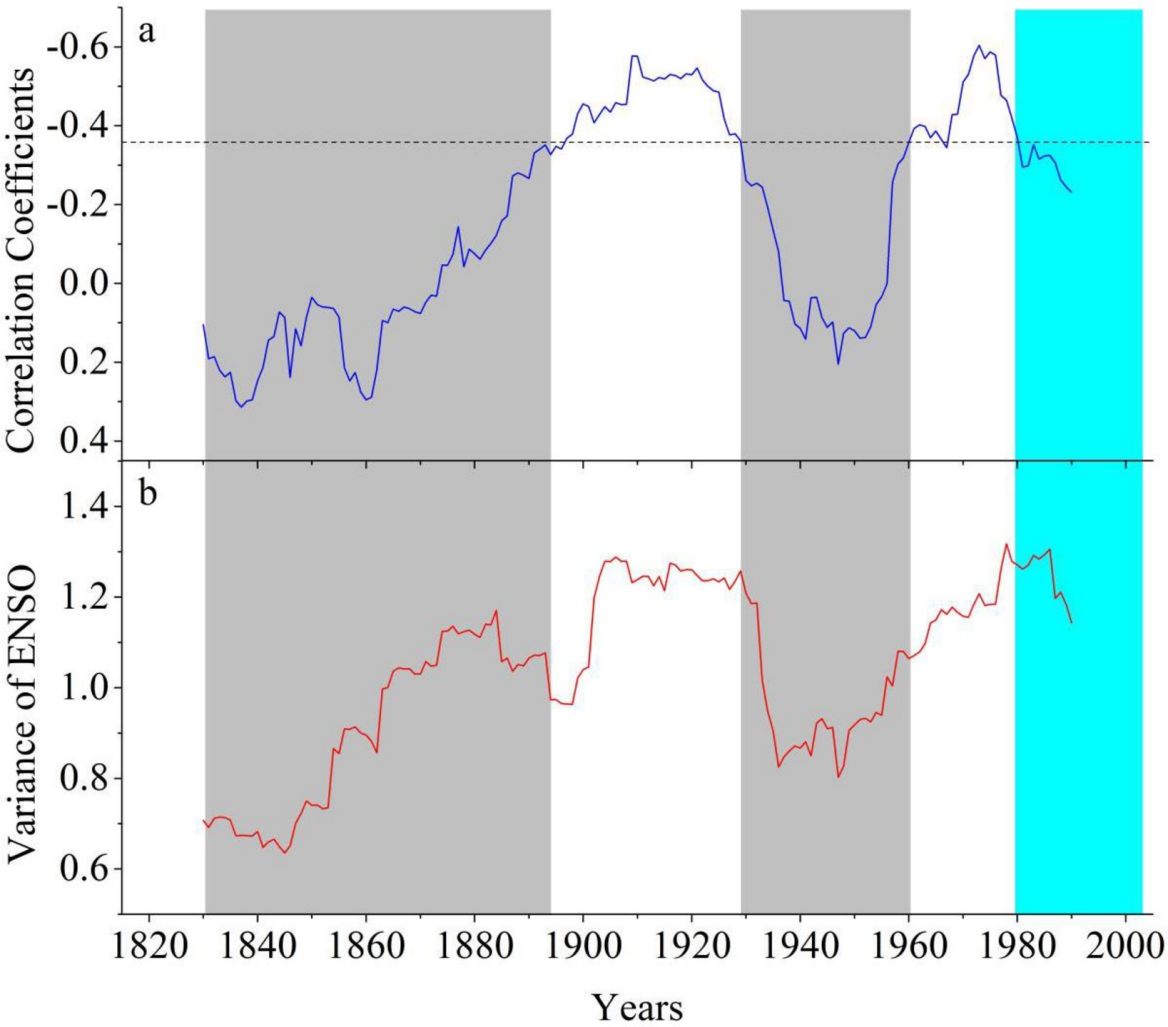
a) The 31-year running correlations (assigned to center year of the window) between the reconstructed EASM and the reconstructed ENSO variability [47] during the common period of 1815–2005. The dashed line symbolizes the 95% confidence level; b) The 31-year running variability of the reconstructed ENSO indices during the period of 1815–2005.

Spatial correlations between the reconstructed EASM and global December-February SSTs show an El Niño-Southern Oscillation (ENSO) like pattern during the common period of 1870–2014 (Fig 5). However, based on the 31-year sliding correlations between the reconstructed EASM and the tree-ring-based ENSO reconstruction [47] during the common period of 1815–2005 (Fig 6A), the EASM-ENSO relationships broke down during the periods of 1830–1890 and 1930–1960 when the ENSO variance was low (Fig 6B).

## Discussion

### Climatic implications of tree-ring δ^18^O

Based on tree-ring cellulose isotope fractionation model, negative correlations are expected between tree-ring cellulose δ^18^O and relative humidity [25]. Low relative humidity enhances vapor pressure gradients between leaf interstitial spaces and the atmosphere, which could cause the preferential loss of lighter isotope and enrich the oxygen isotopes in leaf water [25]. Lower humidity also increases the evaporation of soil water in the upper layer, thus the source water taken up by roots becomes heavier [29]. Similarly, high temperature normally causes enhanced evapotranspiration and decreased relative humidity levels. Therefore, positive correlations are observed between tree-ring cellulose δ^18^O and temperature in the study area.

Negative relationships between precipitation and tree-ring cellulose δ^18^O levels are reported frequently in monsoonal areas [28, 29, 30, 31]. Precipitation affects tree-ring cellulose δ^18^O mainly via relative humidity and δ^18^O in the precipitation itself. On one hand, more precipitation usually equals higher relative humidity during the monsoonal season in the study area (S4 Fig). On the other hand, due to the amount effect [24, 25], isotope-enriched water vapor is more easily removed by condensation due to the mass discrepancy between H_2_^18^O and H_2_^16^O, making the isotopic composition of the remaining vapor lighter [48]. The higher the amount of the precipitation totals, the more depleted is the isotopic composition of the water vapor. Therefore, greater precipitation is associated with higher relative humidity and more δ^18^O depleted precipitation, leading to lower tree-ring cellulose δ^18^O.

### Historical EASM variations

**Fig 7:**
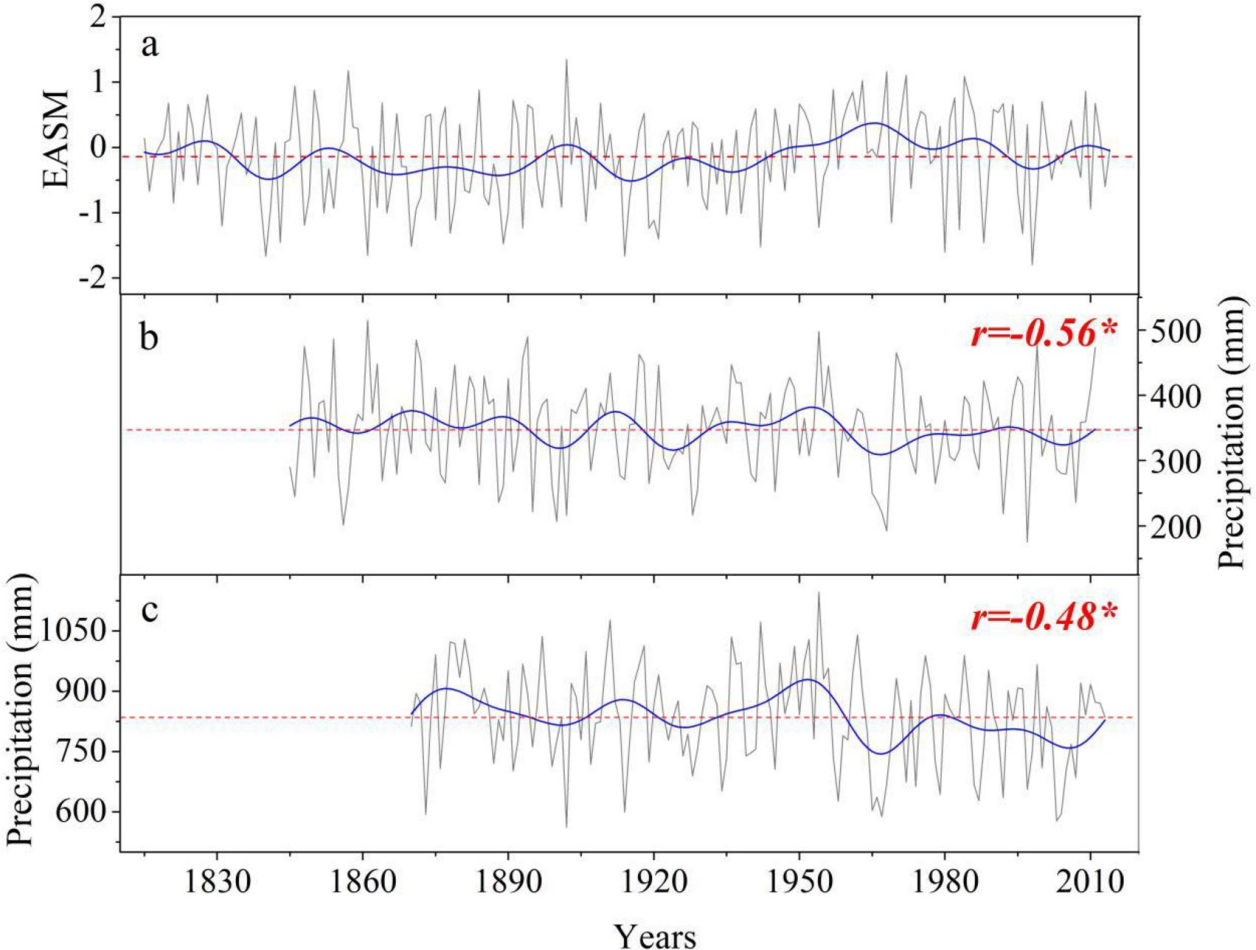
Comparison of (a) the reconstructed EASM with the tree-ring δ^18^O based (b) May-June precipitation reconstruction for BMJ [31] and (c) May-October precipitation reconstruction for AJ [30] over the middle-lower reaches of the Yangtze River. The low-frequency variations isolated by using a 20-year fast-Fourier transform filter are represented by the solid blue lines.

We used tree-ring δ^18^O to reconstruct EASM variations based on the strong linear correlation between tree-ring δ^18^O and the EASM index. The EASM reconstruction was compared with the tree-ring δ^18^O based May-June precipitation reconstruction for Baimajian (BMJ, 31.12°N, 116.18°E) and May-October precipitation reconstruction for Anji (AJ, 30.39°N, 119.43°E) (Fig 1A) over the middle-lower Yangtze River basin, repectively [30, 31]. As shown in Fig 7, the reconstructed EASM and the two reconstructed precipitation records exhibit a high degree of coherency at interdecadal scales, with a significant negative correlation (*p*<0.05) value of -0.56 with the BMJ for 1845–2011, and -0.48 with the AJ for 1870–2013. The correlations are significant (*p*<0.05) after accounting for the effective degrees of freedom due to the low-pass filtering using the modified Chelton method [49, 50, 51]. In addition, many anomalous flooding events over the middle-lower reaches of Yangtze River recorded by historical documents [52] and grain-size variations of deposition from the subaqueous Yangtze River delta [53] are consistent with low extremes of the reconstructed EASM (S2 Table). These results here reinforce the robustness of the reconstruction model.

The reconstructed EASM was generally low and fluctuated moderately in the 19th century, which coincided with the end of the Little Ice Age. In history, the decay of summer monsoon generally occurred during the periods of warmer temperature, and vice versa [1, 54, 55, 56]. More dramatic fluctuations were observed since the 20th century, and the most severe and long-lasting above-average EASM anomalies over the past two centuries occurred during the 1950s-1980s. The stronger EASM during this period may be associated with the increased thermal gradient between the Eurasian continent and the tropical oceans due to global warming, as suggested by a number of synoptic and modeling studies [57, 58, 59]. In addition, although concomitant with global warming, EASM has experienced a moderate decreasing tendency since the 1970s, which is consistent with analysis based on the instrumental data [60, 61]. Despite having mechanisms proposed for the recent weakening of EASM [62, 63, 64, 65], great uncertainties still exist [65]. Some studies attribute the current weakening of EASM to the enhancement of anthropogenic activities [61, 62], while others consider the impacts of natural processes such as oceanic and atmospheric modes [63, 64].

### Links with large-scale atmospheric-oceanic circulations

ENSO acts as one of the most dominant sources in atmosphere-ocean interactions in the tropics, and modulates the climate in extratropical regions through teleconnection [66, 67, 68]. High (low) ENSO variability usually corresponds to strong (weak) ENSO-climate teleconnection [69]. ENSO variance was relatively low during the periods of 1830–1890 and 1930–1960, possibly causing the collapse of the ENSO-EASM teleconnection. When ENSO variance was high, ENSO made significant impacts on EASM changes via modulations of the strength and position of the Western Pacific Subtropical High [70, 71]. Similar interdecadal shifts of ENSO teleconnection have been often highlighted for hydroclimate change over East China and many other regions [28, 29, 67, 68].

Although ENSO still plays an important role for controlling EASM variability, it is worth noting that the strong EASM-ENSO relationship has weakened since 1980s (S6 Fig and Fig 6), raising the possibility that the driving force of EASM dynamics has shifted from natural to anthropogenic in nature [72, 73, 74, 75]. Anthropogenic aerosol emissions in East Asia have increased dramatically since the 1980s because of China’s urbanization and industrialization, which can partially mask greenhouse warming and induce strong large-scale atmospheric circulation changes and regional climate responses [72, 73, 74]. Radiative effects due to increased aerosols (especially black carbon) from pollution over the Asian continent could stabilize regional atmospheric convection and hence to reduce the Asia summer monsoon [72, 76, 77, 78]. In addition, the aerosol-induced cooling trend of the upper tropospheric temperature over East Asia also notably modulates the southward shift of the upper-level westerly jet stream and the northward progression of EASM winds without SST-mediated changes, based upon coupled and atmospheric general circulation models [73, 79].

## Conclusions

We provided the first EASM reconstruction from 1815 to 2014 using a *P*. *massoniana* tree-ring cellulose δ^18^O records in the middle Yangtze River basin, a core area of EASM. EASM started to increase with a peak from the 1950s-80s in response to the warming trends. After the 1970s, EASM started to decay, which may correspond to the enhancement of the anthropogenic influences. ENSO is shown to be a critical forcing of EASM variations. However, the EASM-ENSO relationships collapsed when the ENSO variance became weak. Despite the high ENSO variance in the 1980s, the ENSO-EASM teleconnection still started to decrease, indicating the overwhelming effect of the anthropogenic factors over natural processes in more recent years.

## Supporting information

S1 Fig Monthly mean temperature (circle), monthly total precipitation (bar) records and monthly mean relative humidity (square) at the Shimen meteorological station as averaged during 1960–2014.
(DOCX)

S2 Fig The tree-ring standard width chronology of *P*. *massoniana* (green line) at the study area and the sample replication (gray bar). Dash line denotes the year (1815) with SSS>0.85.
(DOCX)

S3 Fig Tree-ring cellulose δ^18^O data of four individual cores from four different trees (WL02, WL19, HP06, HP13) for the period of 1950–2014, respectively. (DOCX)

S4 Fig Relationship between June-August total precipitation and average relative humidity for the period of 1960–2014. (DOCX)

S5 Fig Wavelet power spectrum of the reconstructed EASM. The thick black contour designates 5% significance level against red noise.
(DOCX)

S6 Fig Spatial correlations between EASM and precedent winter (from December to February) global SSTs for the period of 1981–2017. Correlations not significant at the 95% level have been masked out. *The maps were produced from* *https*:*//climexp*.*knmi*.*nl*.
(DOCX)

S1 Table Correlations of the tree-ring cellulose δ18O data shown in S3 Fig. (DOCX)

S2 Table Documented anomalously flooding events in the middle-lower reaches of Yangtze River with weak EASM detected by tree-ring δ18O records. The average value and standard deviation of the reconstructed EASM are -0.12 and 0.71.
(DOCX)
