# Supplementary material for: A tree-ring δ18O based reconstruction of East Asia summer monsoon over the past two centuries
Source: PLoS One. 2020 Jun 9;15(6):e0234421. doi: 10.1371/journal.pone.0234421 (PMC7282632; doi:10.1371/journal.pone.0234421)
Supplement: S1 Fig — Monthly mean temperature (circle), monthly total precipitation (bar) records and monthly mean relative humidity (square) at the Shimen meteorological station as averaged during 1960–2014. (DOCX) [file pone.0234421.s001.docx]

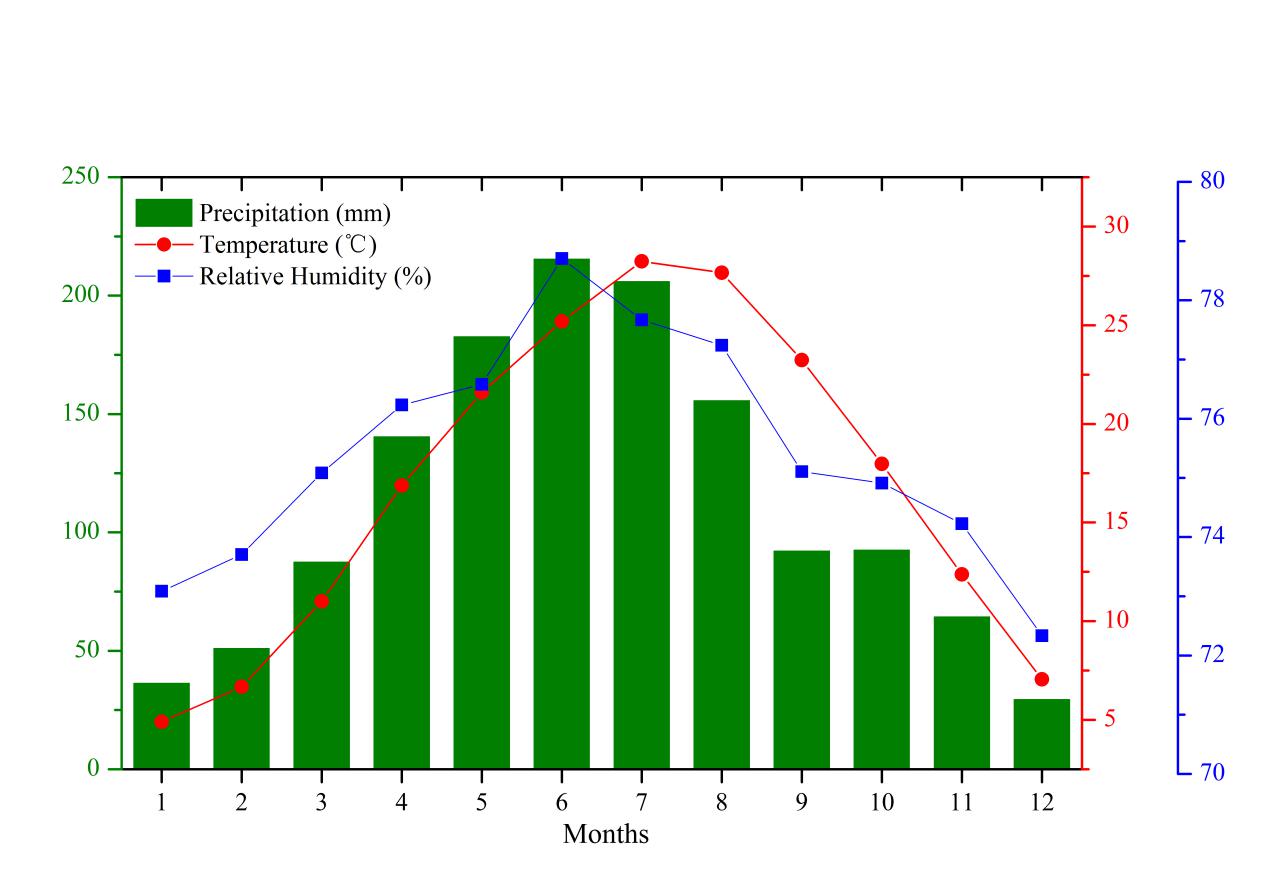


**Fig. S1** Monthly mean temperature (circle), monthly total precipitation (bar) records and monthly mean relative humidity (square) at the Shimen meteorological station as averaged during 1960-2014.
