# Supplementary material for: A tree-ring δ18O based reconstruction of East Asia summer monsoon over the past two centuries
Source: PLoS One. 2020 Jun 9;15(6):e0234421. doi: 10.1371/journal.pone.0234421 (PMC7282632; doi:10.1371/journal.pone.0234421)
Supplement: S2 Fig — The tree-ring standard width chronology of P. massoniana (green line) at the study area and the sample replication (gray bar). Dash line denotes the year (1815) with SSS>0.85. (DOCX) [file pone.0234421.s002.docx]

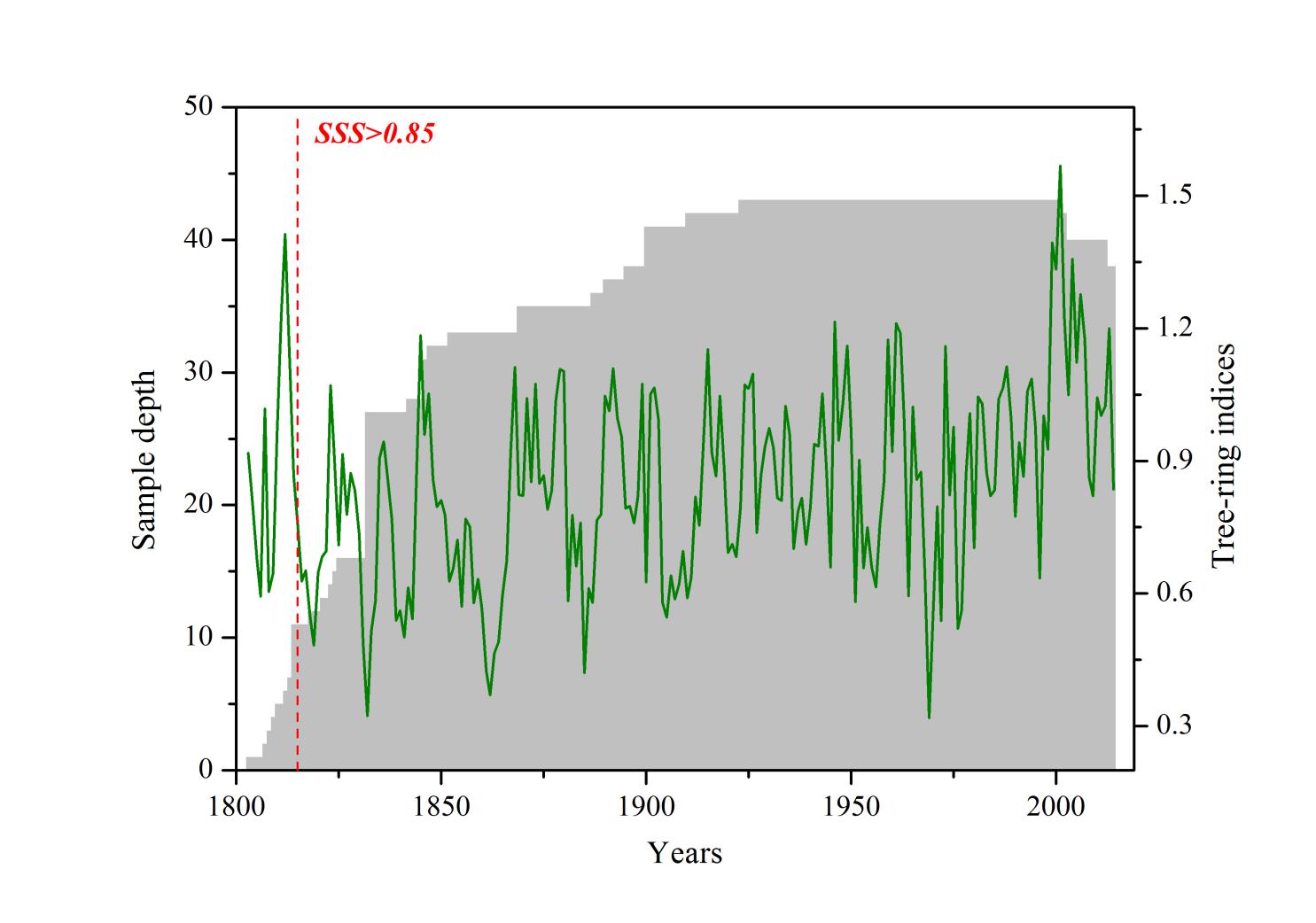


**Fig. S2** The tree-ring standard width chronology of *P. massoniana* (green line) at the study area and the sample replication (gray bar). Dash line denotes the year (1815) with SSS>0.85
