# Supplementary material for: A tree-ring δ18O based reconstruction of East Asia summer monsoon over the past two centuries
Source: PLoS One. 2020 Jun 9;15(6):e0234421. doi: 10.1371/journal.pone.0234421 (PMC7282632; doi:10.1371/journal.pone.0234421)
Supplement: S3 Fig — (DOCX) [file pone.0234421.s003.docx]

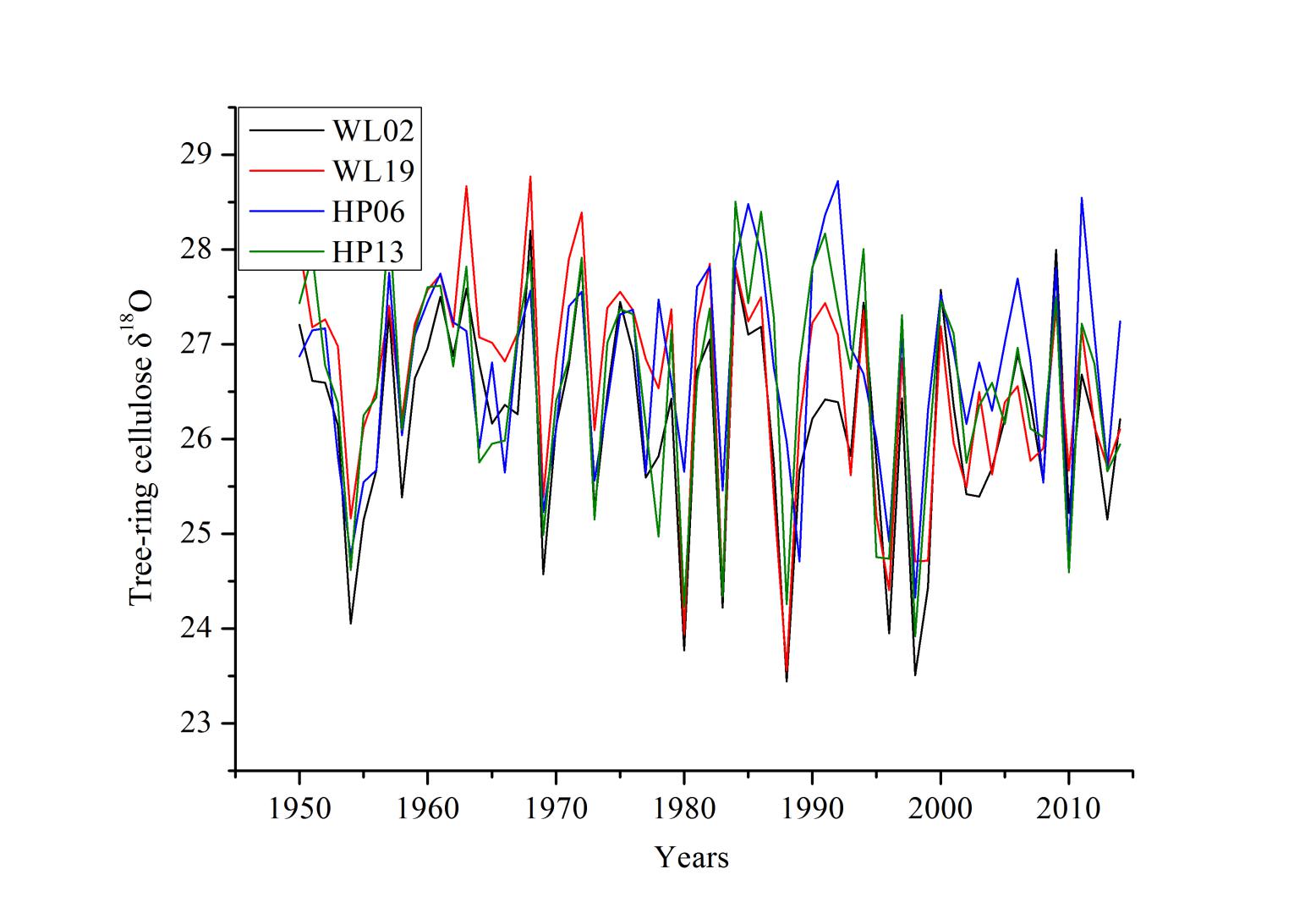


**Fig. S3** Tree-ring cellulose δ^18^O data of four individual cores from four different trees (WL02, WL19, HP06, HP13) for the period of 1950-2014, respectively.
