# Supplementary material for: A tree-ring δ18O based reconstruction of East Asia summer monsoon over the past two centuries
Source: PLoS One. 2020 Jun 9;15(6):e0234421. doi: 10.1371/journal.pone.0234421 (PMC7282632; doi:10.1371/journal.pone.0234421)
Supplement: S4 Fig — (DOCX) [file pone.0234421.s004.docx]

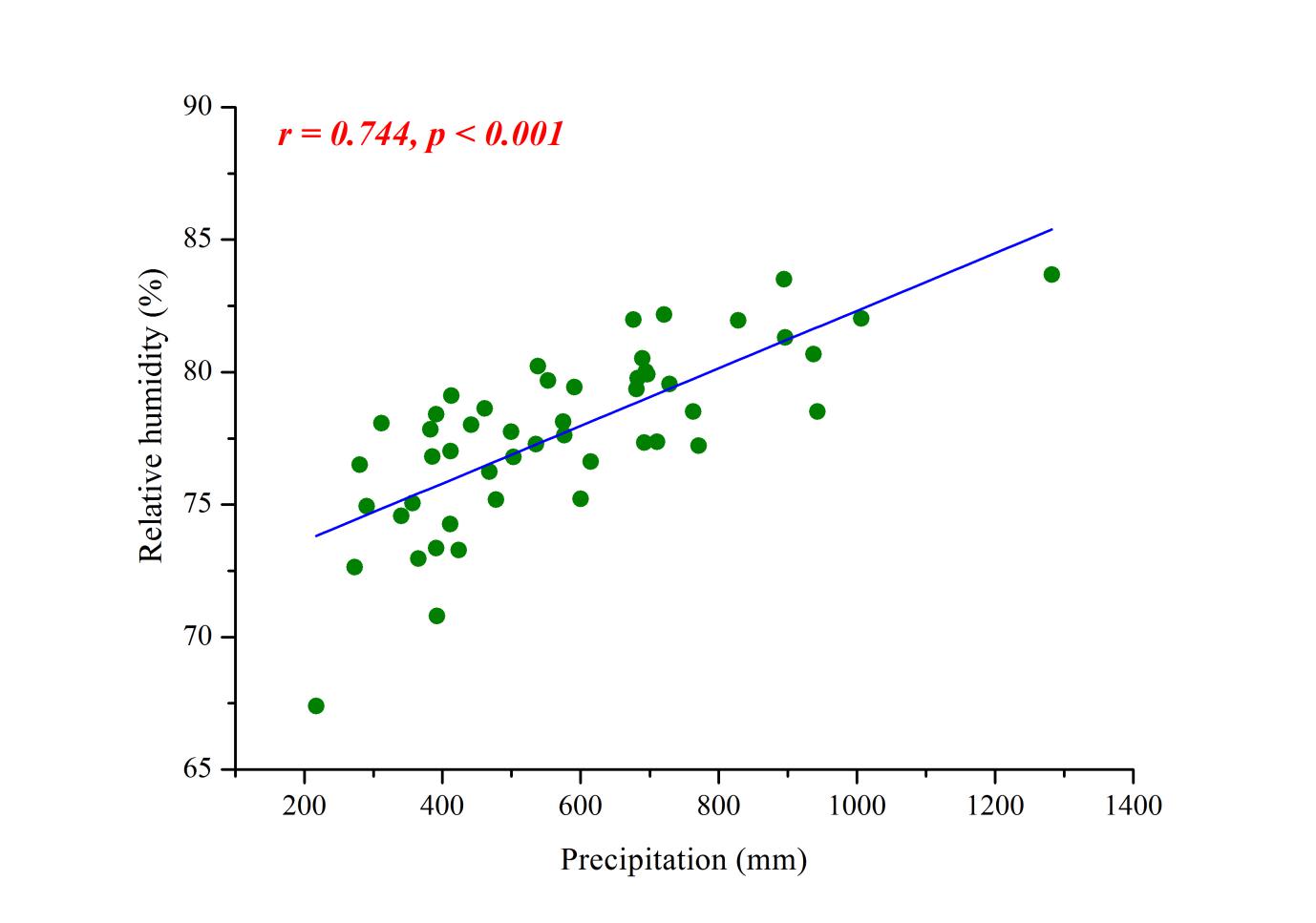


**Fig. S4** Relationship between June-August total precipitation and average relative humidity for the period of 1960-2014.
