# Supplementary material for: A tree-ring δ18O based reconstruction of East Asia summer monsoon over the past two centuries
Source: PLoS One. 2020 Jun 9;15(6):e0234421. doi: 10.1371/journal.pone.0234421 (PMC7282632; doi:10.1371/journal.pone.0234421)
Supplement: S5 Fig — The thick black contour designates 5% significance level against red noise. (DOCX) [file pone.0234421.s005.docx]

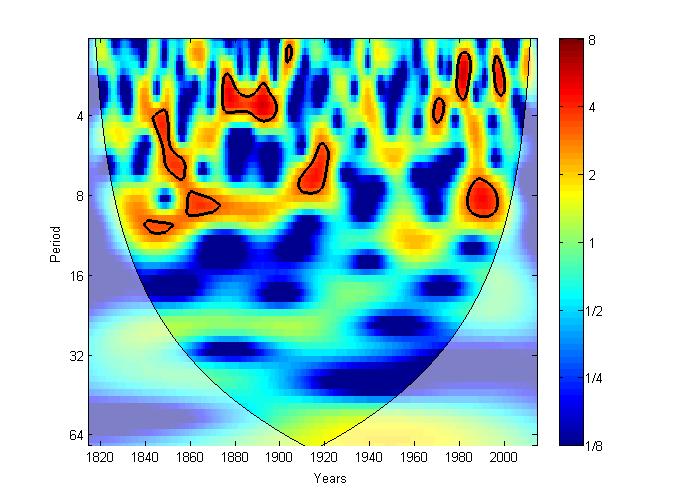


**Fig. S5** Wavelet power spectrum of the reconstructed EASM. The thick black contour designates 5% significance level against red noise.
