# Supplementary material for: A tree-ring δ18O based reconstruction of East Asia summer monsoon over the past two centuries
Source: PLoS One. 2020 Jun 9;15(6):e0234421. doi: 10.1371/journal.pone.0234421 (PMC7282632; doi:10.1371/journal.pone.0234421)
Supplement: S6 Fig — Correlations not significant at the 95% level have been masked out. The maps were produced from https://climexp.knmi.nl. (DOCX) [file pone.0234421.s006.docx]

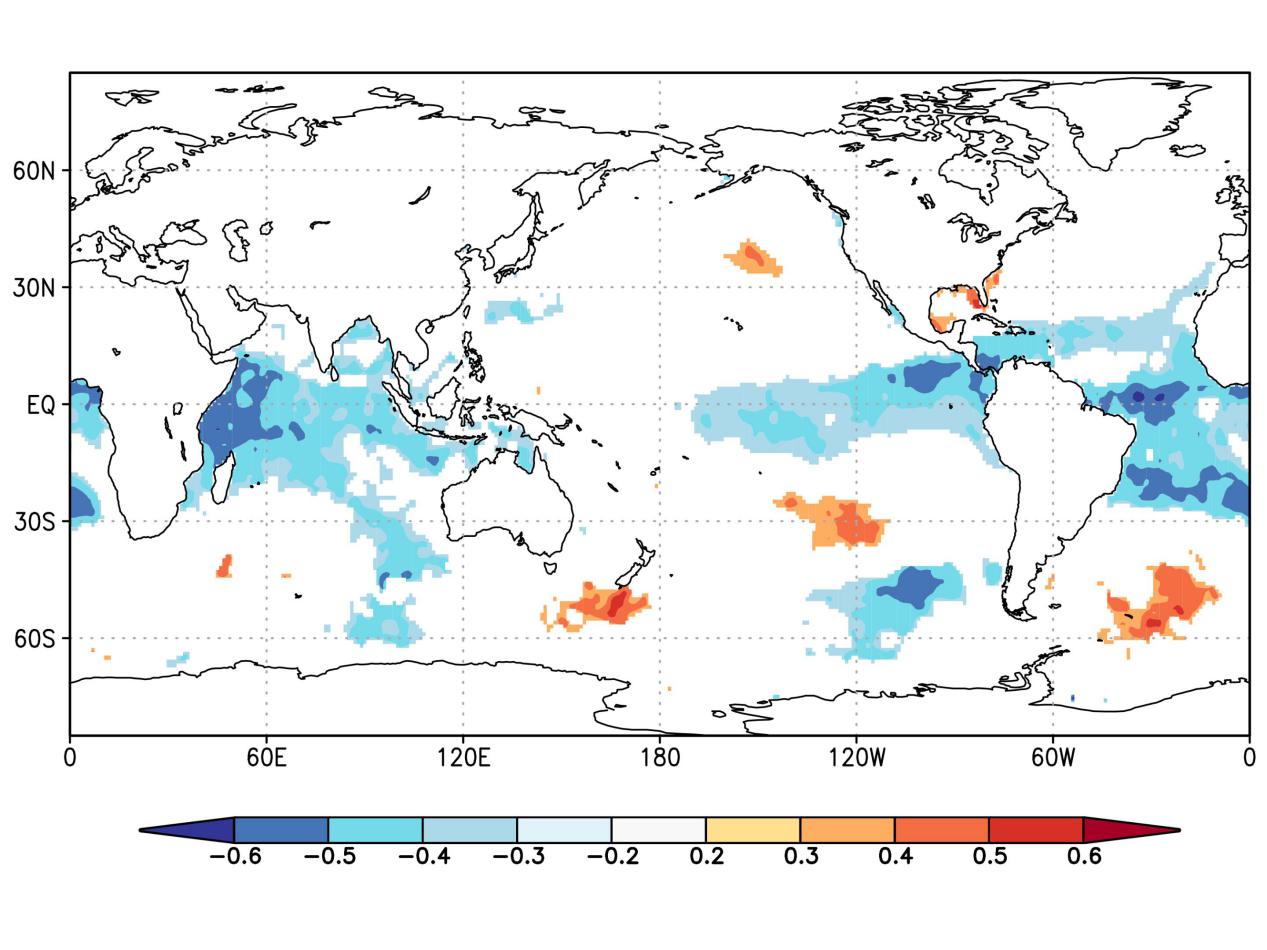


**Fig. S6** Spatial correlations between EASM and precedent winter (from December to February) global SSTs for the period of 1981-2017. Correlations not significant at the 95% level have been masked out. *The maps were produced from* [*https://climexp.knmi.nl*](https://climexp.knmi.nl)*.*
