# Supplementary material for: A tree-ring δ18O based reconstruction of East Asia summer monsoon over the past two centuries
Source: PLoS One. 2020 Jun 9;15(6):e0234421. doi: 10.1371/journal.pone.0234421 (PMC7282632; doi:10.1371/journal.pone.0234421)
Supplement: S1 Table — (DOCX) [file pone.0234421.s007.docx]

**Table S1** Correlations of the tree-ring cellulose δ^18^O data shown in **Fig. S3**.

| r (n=65) | WL19 | HP06 | HP13 |
| --- | --- | --- | --- |
| WL02 | 0.91** | 0.76** | 0.86** |
| WL19 |  | 0.69** | 0.82** |
| HP06 |  |  | 0.78** |

** symbolizes 95% confidence level.
