# Supplementary material for: A tree-ring δ18O based reconstruction of East Asia summer monsoon over the past two centuries
Source: PLoS One. 2020 Jun 9;15(6):e0234421. doi: 10.1371/journal.pone.0234421 (PMC7282632; doi:10.1371/journal.pone.0234421)
Supplement: S2 Table — The average value and standard deviation of the reconstructed EASM are -0.12 and 0.71. (DOCX) [file pone.0234421.s008.docx]

**Table S2** Documented anomalously flooding events in the middle-lower reaches of Yangtze River with weak EASM detected by tree-ring δ^18^O records. The average value and standard deviation of the reconstructed EASM are -0.12 and 0.71.

| Flooding years | EASM strength | Descriptions in the historical documents |
| --- | --- | --- |
| 1831 | -1.23 | Floods inundated many counties in Changde City, Hunan Province; tens of thousands of people were killed. |
| 1840 | -1.66 | [Catastrophic flood](http://www.youdao.com/w/catastrophic%20flood/)s in the upper-middle reaches of Yangtze River. |
| 1848 | -1.19 | More than 100 counties were hit by disastrous flooding in Hubei, Hunan, Jiang Xi, Anhui and Jiangsu. |
| 1869 | -1.30 | More than 100 counties were hit by disastrous flooding over the middle-lower  reaches of Yangtze River. |
| 1870 | -1.51 | The peak discharge of Yichang hydrologic  station reached 10,500 m³/s, the highest  record in the history. |
| 1889 | -1.48 | Great flooding hit Hubei, Anhui, Jiangsu and  Zhejiang, especially the Taihu Lake basin. |
| 1906 | -1.13 | [Catastrophic flood](http://www.youdao.com/w/catastrophic%20flood/) occurred in Xiangjiang River, Hunan Province, causing 30,000~40,000 people death. |
| 1921 | -1.39 | Floods hit Hunan, Hubei, Jiangsu and Anhui,  resulting in thousands of people death. |
| 1931 | -1.26 | June-September average discharge anomaly of Hankou hydrologic station was 13,700 m³/s; 145,000 people were killed. |
| 1935 | -1.02 | June-September average discharge anomaly of Hankou hydrologic station was 6,800 m³/s; nearly 142,000 people were killed. |
| 1954 | -1.22 | The peak discharges of Yichang and Hankou hydrologic stations reached 66,800 m³/s and 76,100 m³/s; more than 30,000 peoplewere dead. |
| 1969 | -1.15 | The hardest-hit areas by floods were Hubei  and Anhui; More than 30,000 people were  killed. |
| 1980 | -1.60 | The peak discharge of Hanjiang River was 60,100 m³/s; over 10,000 people were killed in Hunan, Anhui, Jiangxi and Hubei. |
| 1983 | -1.26 | The peak discharges of Hankou hydrologic  stations reached 65,000 m³/s. |
| 1996 | -1.32 | The peak discharges of Hankou hydrologic stations was 55,900 m³/s. |
| 1998 | -1.80 | The entire Yangtze River basin was hit by an catastrophic flood; the peak discharges ofHankou hydrologic stations was 71,100 m³/s. |
